# cTNM vs. pTNM: the effect of not applying ultrasonography in the identification of cervical nodal disease

**DOI:** 10.1186/1758-3284-4-5

**Published:** 2012-03-12

**Authors:** Waseem Jerjes, Tahwinder Upile, Hani Radhi, Aviva Petrie, Jesuloba Abiola, Aidan Adams, Jacqueline Callear, Panagiotis Kafas, Syedda Abbas, Kartic Rajaram, Colin Hopper

**Affiliations:** 1 UCL Department of Surgery, University College London, London, UK; 2 Oral and Maxillofacial Surgery Unit, AL-Mustansirya University, Baghdad, Iraq; 3 Leeds Institute of Molecular Medicine, School of Medicine, University of Leeds, Leeds, UK; 4 Chase Farm & Barnet NHS Trust, Enfield, UK; 5 Head & Neck Unit, University College London Hospital, London, UK; 6 Biostatistics Unit, UCL Eastman Dental Institute, London, UK; 7 Department of Oral Surgery and Radiology, School of Dentistry, Aristotle University, Thessalonica, Greece

## Abstract

Accurate clinical staging of oral squamous cell cancer can be quite difficult to achieve especially if nodal involvement is identified. Radiologically-assisted clinical staging is more accurate and informs the clinician of loco-regional and distant metastasis.

In this study, we compared clinical TNM (cTNM) staging (not including ultrasonography) to pathological TNM (pTNM) staging in 245 patients presenting with carcinoma of the oral cavity and the oro-pharyngeal region. Tumour size differences and nodal involvement were highlighted. US reports of the neck were then added to the clinical staging and results compared.

Tumour size was clinically underestimated in 4 T1, 2 T2 and 2 T3 oral diseases. Also 20 patients that were reported as nodal disease free had histological proven N1 or N2 nodal involvement; while 3 patients with cTNM showing N1 disease had histologically proven N2 disease.

Overall the agreement between the 2 systems per 1 site was 86.6% (Kappa agreement = 0.80), per 2 sites 90.0% (Kappa agreement = 0.68) and per 3 sites 90.5% (Kappa agreement 0.62).

An accurate clinical staging is of an utmost importance. It is the corner stone in which the surgical team build the surgical treatment plan and decide whether an adjuvant therapy is required to deal with any possible problem that might arise. The failure to achieve an accurate staging may lead to incomplete surgical planning and hence unforeseen problems that may adversely affect the patient's survival.

## Background

The development of modern imaging techniques has significantly altered the treatment and management of head and neck malignancies. Important treatment decisions that were once made intra-operatively are now made preoperatively by means of advanced imaging, i.e. computed tomography (CT) and magnetic resonance imaging (MRI) [1].

MRI has been proved superior to CT, on account of its coronal and sagittal slice orientation enabling not only a better demonstration of findings, but also improved tumour detection and staging [2]. Also when it comes to T-staging, MRI is overall more accurate than CT. However, if degraded images and T1 tumours are excluded, the techniques are comparable. MRI is oversensitive for recurrent disease. For Nodal staging, MRI was found comparable to CT [3]. Although CT and MRI allow detection of abnormally enlarged nodes or necrotic nodes, neither borderline-sized nodes without necrosis nor extra-capsular spread are reliably differentiated from reactive or normal nodes in patients with head and neck cancer [4].

Accurate clinical staging of pathologies involving the oral and oro-pharyngeal-laryngeal regions can be quite difficult to achieve, especially if nodal disease is involved. The current practice worldwide when it comes to clinically staging tumours identified in these areas usually involve carrying out a thorough clinical examination of the head and neck region followed by radiological investigations. The latter usually involve carrying out an MRI of the head and neck, or a CT scanning if bony invasion is anticipated (i.e. edentulous patients). The rest of the clinical staging involve a CT scanning of the chest and the upper abdomen to rule out any tumour spread to the pulmonary or hepato-pancreato-biliary organs [5].

In most of the centres in the developed world, an ultrasonography (US) imaging of the neck is carried out to identify any nodal involvement which cannot be identified by other imaging modalities (i.e. MRI or CT); unfortunately the lack of expertise in the developing world stops the application of US technology. US is a reliable and valuable tool for metastatic lymph node screening in head and neck cancer patients. It is a cheap, non-invasive, easy-to-handle and cost-effective diagnostic method [6].

This has lead many centres around the world to change its practice to include US as part of clinical staging process for any patient who presents with pathological lesion in the head and neck. Furthermore, the advances in cytological sciences allowed fine needle aspiration of neck nodes under US-guidance and allowed identification of reactive lymph nodes prior to surgery. A study identified US, with or without FNAC, as an accurate (86%), sensitive (92%) and specific (83%) technique for the preoperative assessment of lymph node metastases in patients with SCC [7]. In another study, US (for all levels) yielded a sensitivity of 71%, and a specificity of 87%, while CT showed a sensitivity of 32% and a specificity of 96%. The sensitivity of US decreased from level I to level IV, whereas the specificity increased from level I to level IV [8].

In this short communication, we retrospectively compared clinical TNM (cTNM) staging (not including US) to pathological TNM (pTNM) staging in patients presenting with carcinoma of the oral cavity and the oro-pharyngeal region. Tumour size differences and nodal involvement were highlighted. US reports of the neck were then added to the clinical staging and results compared.

## Materials & methods

In this retrospective analytic study, we looked at cTNM staging and pTNM staging of 245 patients with oral and pharyngeal squamous cell carcinoma who underwent treatment at the UCLH Head and Neck Centre, London from 2001 to 2006.

The patients' data were entered onto proformas, which were validated and checked by interval sampling. The fields included a range of clinical, operative and histo-pathological variables related to disease staging. All applications were accompanied by multidisciplinary team recommendation, ethical approval and informed patient consent.

In this short communication, the cTNM staging was achieved through clinical examination of the head and neck region and MRI, or CT scan when indicated, and no US scan of the neck. This was compared to pTNM trying to identify any underestimation in tumour size or missed cervical nodal disease. Agreement between both staging systems were assessed per anatomical site. Then, US reports were added to the cTNM staging and results compared again.

### Statistical analysis

The outcomes of the categorical clinic-pathological variables (cTNM and pTNM) were summarised as frequencies and percentages for the whole group of patients. Kappa score agreement, with its standard error, was calculated to compare cTNM and pTNM for primary, secondary and tertiary sites; also was applied to per 1 site, per 2 sites and per 3 sites.

## Results

Table 1 highlights the cTNM and the pTNM staging of the cohort involved in this study. Tumour size was clinically underestimated in 4 T1, 2 T2 and 2 T3 oral diseases. Also 20 patients that were reported as nodal disease free had histological proven N1 or N2 nodal involvement; while 3 patients with cTNM showing N1 disease had histologically proven N2 disease (Table 1). When the US data was added to the cTNM staging, no differences were identified in nodal disease assessment.

**Table 1:** The cTNM and the pTNM staging of the cohort involved in this study

	cTNM (with no US) Frequency (%)	pTNM Frequency (%)	Tumour size underestimated	Nodal disease missed
T1N0M0	107 (43.7)	96 (39.2)	2 (T2)	5 (N1), 3 (N2)
T2N0M0	36 (14.7)	32 (13.1)	1 (T3)	5 (N1), 1 (N2)
T3N0M0	20 (8.2)	18 (7.3)	2 (T4)	1 (N1)
T4N0M0	45 (18.4)	42 (17.1)	-	4 (N1), 1 (N2)
T1N1M0	10 (4.1)	13 (5.3)	2 (T2)	
T2N1M0	12 (4.9)	16 (6.5)	1 (T3)	1 (N2)
T3N1M0	9 (3.7)	10 (4.1)	-	2 (N2)
T4N1M0	6 (2.4)	10 (4.1)	-	-
T1N2M0	0 (0.0)	3 (1.2)	-	-
T2N2M0	0 (0.0)	2 (0.8)	-	-
T3N2M0	0 (0.0)	2 (0.8)	-	-
T4N2M0	0 (0.0)	1 (0.4)	-	-

The table also identifies the number of patients with underestimated tumour size and missed nodal disease

Agreement on anatomical sites between cTNM and pTNM, as well as kappa scores, are highlighted in Table 2. The agreement between cTNM (without US) and pTNM when assessing the tongue was 95.7%, FOM 86.6%, buccal mucosa 82.9%, retromolar area 75.0% and tonsils 91.7%. This may be due to the fact that deep and posterior oral structures are a challenge during clinical examination as well as the dental artifacts that could arise during scanning which affects staging of peri-dental tumours (Table 2).

**Table 2:** Agreement on anatomical sites between cTNM and pTNM, as well as kappa scores

		Total	Agreement	Kappa	Std. Err of kappa
Primary	Tongue	47	95.74%	0.94	0.07
	FOM	82	86.59%	0.82	0.05
	Retromolar area	12	75.00%	0.69	0.12
	Buccal mucosa	70	82.86%	0.77	0.06
	Lower lip	7	85.71%	0.79	0.20
	Gingiva	7	85.71%	0.81	0.20
	Hard palate	3	100.00%	1.00	0.58
	Soft palate	4	100.00%	1.00	0.36
	Tonsils	12	91.67%	0.88	0.17
	**Overall**	**245**	**87.35%**	**0.83**	**0.03**
Secondary	Tongue	12	75.00%	0.00	0.00
	FOM	15	100.00%	1.00	0.26
	Gingiva	16	93.75%	0.82	0.25
	Tonsils	3	66.67%	0.00	-
Tertiary	Buccal mucosa	6	100.00%	1.00	0.41
	Gingiva	7	71.43%	0.00	0.00
Per 1 site	-	194	86.60%	0.80	0.04
Per 2 sites	-	30	90.00%	0.68	0.16
Per 3 sites	-	21	90.48%	0.62	0.20

Overall the agreement between the 2 systems per 1 site was 86.60% (Kappa agreement = 0.80), per 2 sites 90.0% (Kappa agreement = 0.68) and per 3 sites 90.48% (Kappa agreement 0.6182), (Table 2).

## Discussion

Cancers of the oral cavity and oro-pharyngeal region are the sixth most common cancers in the world. Unfortunately the incidence continues to rise with moderate survival rates, despite the recent advances in surgery and radiotherapy [9-11]. Oral squamous cell carcinoma (OSCC) continues to affect more males than females with a ratio of 1.5-1. Diagnosis is usually at the fifth or sixth decade of life. However there is increase in the trend of oral cancer affecting young people under the age of 45 years reaching to about 6%. Oral cancer has been found to be higher in ethnic minorities in other developed countries [9-11]. The most common oral sites to be affected with SCC include the lateral border of the tongue, ventral tongue and floor of mouth. In the Asian population, the buccal mucosa is commonly affected due to betel quit/tobacco chewing habits [9-11].

The tumour size is one of the most important factors affecting prognosis. This usually affects the clinician's ability to decide between ablative surgery, radiotherapy, chemotherapy, photodynamic therapy or just proceed to palliative care which could include a combination of any of the above therapies. Also, it is well documented that increased tumour size is related to local and regional disease spread, higher recurrence rates and poor prognosis [11-15].

Loco-regional spread to the cervical chain complicates treatment options and worsen the outcome. Several factors have been known to influence local and regional tumour spread to the lymphatics and they include tumour primary site and thickness, double DNA aneuploidy, poorly differentiated tumours, infiltrating-type invasive front and perineural and peri/endovascular invasion. Distant tumour spread occurs most commonly in uncontrolled local and regional disease and nodal disease [11-15].

The influence of the histological grading as a prognostic factor in oral squamous cell carcinoma have been assessed in several studies and found to be a significant predictor of local and regional failure as well as tumour recurrence. Positive close tumour margins are usually associated with high risk of local recurrence and have a negative effect on survival [11-15].

An accurate clinical staging is of an utmost importance. It is the corner stone in which the surgical team build the surgical treatment plan and decide whether an adjuvant therapy is required to deal with any possible problem that might arise (i.e. impossible to achieve negative margins near vital neurovascular structure or managing distant disease causing pulmonary haemorrhage prior to surgery). The failure to achieve an accurate staging may lead to incomplete surgical planning and hence unforeseen problems that may adversely affect the patient, leading to higher morbidity and mortality.

It is debatable that inaccurate registration of nodal involvement during the clinical TNM staging is unlikely to affect the patient's prognosis. Histo-pathological grading would rectify the error and the patient would undergo an adjuvant therapy (i.e. radiation) that would be delivered anyway in the postoperative phase.

In this short communication, we highlighted the significant differences between cTNM and pTNM when US is not used in assessing for cervical nodal disease.

## Competing interests

The authors declare that they have no competing interests.

## Authors' contributions

All authors have contributed intellectually and to the writing of this manuscript. AP: contributed to the statistical analysis of this study. All authors read and approved the final manuscript.
